# A Man With Exertional Dyspnea

**DOI:** 10.1016/j.acepjo.2025.100156

**Published:** 2025-05-05

**Authors:** Jumpei Yamashita, Daisuke Mizu, Hidenori Higashi, Masataka Miyamoto

**Affiliations:** Department of Emergency Medicine, Japanese Red Cross Osaka Hospital, Osaka, Japan

**Keywords:** vanishing lung syndrome, pneumothorax, lung ultrasonography

## 1 Patient Presentation

**Figure 1:**
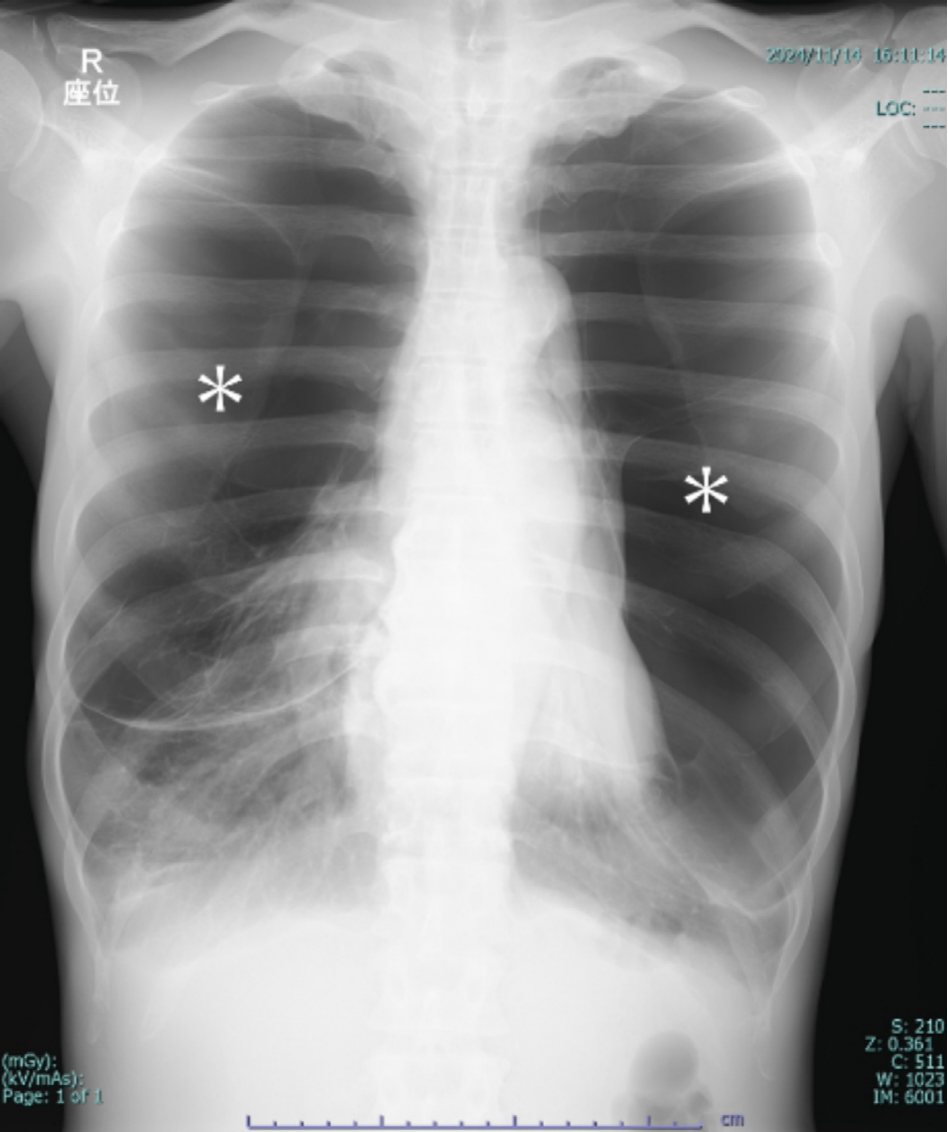
Chest radiography showing increased lung translucency and absent vascular markings in the upper and middle lung fields bilaterally (asterisks).

**Figure 2:**
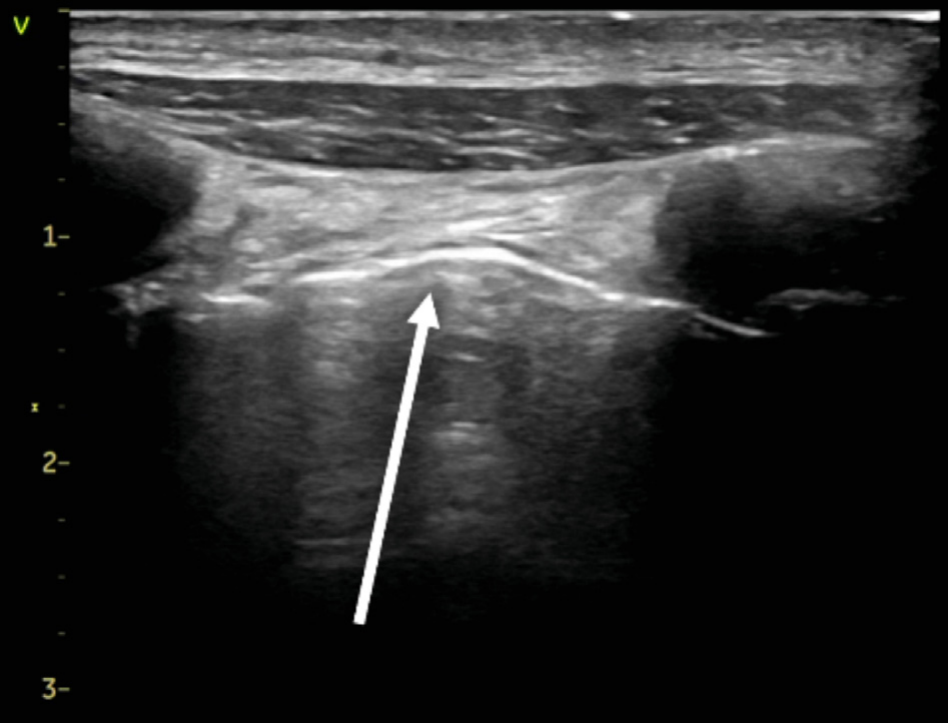
Lung ultrasonography showing sliding signs (arrow).

**Figure 3:**
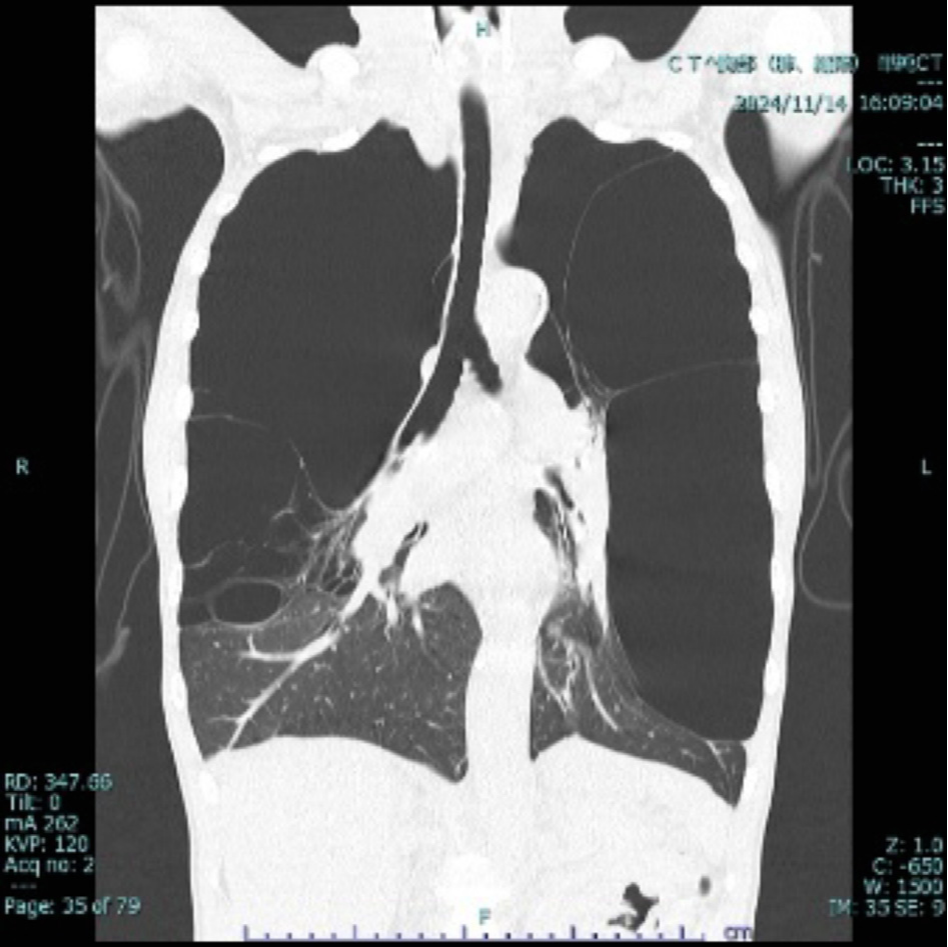
The area of hyperlucency that appeared to be a pneumothorax on chest radiography is found to be a giant bulla on computed tomography.

A 48-year-old man with a history of smoking (30 cigarettes per day for 30 years) presented to our emergency department with exertional dyspnea. Bilateral respiratory sounds were weakened, the respiratory rate was 22, and the oxygen saturation level was 90% on ambient air. Chest radiography revealed increased lung translucency bilaterally (Fig 1). Although we initially suspected bilateral pneumothorax, lung ultrasonography revealed a lung sliding sign (Fig 2; Video), ruling out pneumothorax. Computed tomography revealed giant bullae with compressed lung parenchyma (Fig 3).

**Video:**
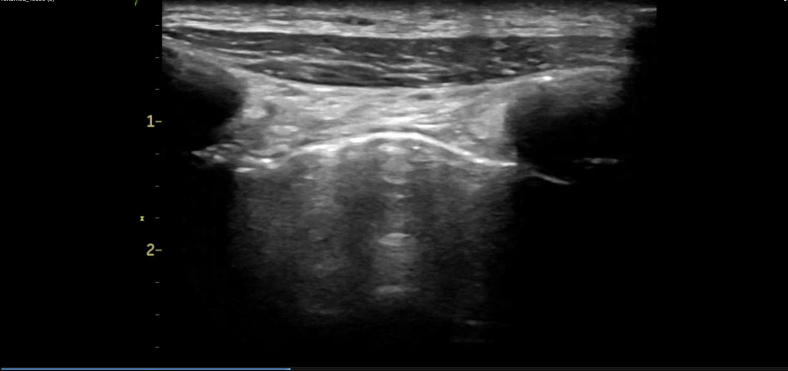
Lung sliding signs showing vanishing lung syndrome.

## 2 Diagnosis: Vanishing Lung Syndrome With Idiopathic Giant Bullous Emphysema

Vanishing lung syndrome (VLS) is characterized by giant bullae occupying more than one-third of the thoracic cavity, compressing surrounding lung tissue.^1^ It typically originates in the upper lobes.^1^ Radical treatment involves surgical resection,^2^^,^^3^ and the patient underwent bullectomy under veno-venous extracorporeal membrane oxygenation support at a later date. VLS presents with diminished breath sounds and increased lung translucency on radiography, mimicking pneumothorax. In patients with VLS, placing a chest tube may cause secondary pneumothorax.^1^^,^^4^ In the present case, lung ultrasonography revealed a sliding sign, which led us to rule out pneumothorax and prevent unnecessary drainage. This case highlights the use of lung ultrasonography as an immediate diagnostic tool for differentiating VLS from pneumothorax.

## Funding and Support

By *JACEP Open* policy, all authors are required to disclose any and all commercial, financial, and other relationships in any way related to the subject of this article as per ICMJE conflict of interest guidelines (see www.icmje.org). The authors have stated that no such relationships exist.

## Conflict of Interest

All authors have affirmed they have no conflicts of interest to declare.
